# Over Two Decades of Experience in Aortic Arch Reoperations: Long-Term Outcomes and Mortality Risk Factors

**DOI:** 10.3390/jcm14124087

**Published:** 2025-06-10

**Authors:** Nikoleta Bozini, Nicole Piber, Keti Vitanova, Konstantinos Sideris, Ulf Herold, Ralf Guenzinger, Andrea Amabile, Teodora Georgescu, Markus Krane, Anatol Prinzing

**Affiliations:** 1 Department of Cardiovascular Surgery, Institute Insure, German Heart Center Munich, School of Medicine & Health, Technical University of Munich, Lazarettstrasse 36, 80636 Munich, Germany; bozini@dhm.mhn.de (N.B.); piber@dhm.mhn.de (N.P.); vitanova@dhm.mhn.de (K.V.); sideris@dhm.mhn.de (K.S.); herold@dhm.mhn.de (U.H.); guenzinger@dhm.mhn.de (R.G.); amabile.andrea@gmail.com (A.A.); georgescu@dhm.mhn.de (T.G.); 2 Division of Cardiac Surgery, Department of Cardiothoracic Surgery, University of Pittsburgh, Pittsburgh, PA 15213, USA; 3 UPMC Heart and Vascular Institute, University of Pittsburgh Medical Center, Pittsburgh, PA 15213, USA; 4 DZHK (German Center for Cardiovascular Research)—Partner Site Munich Heart Alliance, 80636 Munich, Germany; 5 Department of Cardiovascular Surgery, University Hospital and Goethe University Frankfurt, 60323 Frankfurt, Germany; anatol.prinzing@unimedizin-ffm.de

**Keywords:** aortic arch, reoperation, redo, aortic surgery, outcome, mortality

## Abstract

**Background/Objectives:** After years of work in the field of aortic arch surgery, the technique has evolved, making this procedure relatively safe, with lasting results. Due to the increasing long-term survival and overall aging of the patient population, more patients require aortic arch reoperation. In the present study, the safety of aortic arch reoperations was analyzed in the long term, focusing on risk factors for mortality. **Methods:** Between 1999 and 2023, 108 patients were included in our study who underwent reoperation on aortic arch after prior operation on the aorta, the aortic valve, or a combination of both. The exclusion criteria were being aged under 18 years and transcatheter aortic valve implantation as a previous intervention. The principal outcome was the incidence of mortality, and additional outcomes of interest included cardiac re-reoperation, bleeding, a new aortic type B dissection, infective endocarditis, readmission due to a cardiac cause, coronary intervention and neurovascular complications, pacemaker implantation, and temporary mechanical circulatory support. **Results:** The mean age was 56 ± 14 years, and 75% (81/108) of patients were male. In our study, we found age (*p* ≤ 0.01) and history of coronary artery disease (*p* = 0.01) to be preoperative risk factors for adverse outcomes. The mean time between the index operation and reoperation was 6.84 years (1.61–14.94). Indications for reoperation included dilatation (HR = 0.49, *p* = 0.05), rupture or false aneurysm (HR = 2.08, *p*= 0.08), dissection (HR = 1.41, *p* = 0.30), and endocarditis (HR = 1.49, *p* = 0.41). A main risk factor was the need for a salvage reoperation (*p* ≤ 0.01). Also, a longer operation (*p* = 0.04), cardiopulmonary bypass (*p* ≤ 0.01), and ventilation time (*p* ≤ 0.01), bleeding complications (*p* ≤ 0.01), and requiring temporary mechanical circulatory support (*p* = 0.04) were linked to higher mortality. The overall survival was 82% after 1 year, 73% after 5 years, and 56% after 10 years. In the multivariate Cox regression analysis, age (HR = 1.04, *p* ≤ 0.01), the need for a salvage operation (HR = 5.38, *p* = 0.01), a prolonged ventilation time (HR = 1.08, *p* = 0.04), and bleeding complications (HR = 3.76, *p* = 0.03) were associated with higher mortality. In the ROC analysis, an age over 57.5 years was associated with significantly lower overall survival (*p* ≤ 0.01). **Conclusions:** Aortic arch reoperations can be performed with acceptable long-term outcomes, but perioperative factors significantly influence early mortality. Salvage operations, bleeding complications, and prolonged ventilation were strong predictors of adverse outcomes. Older age, particularly >57.5 years, was independently associated with increased mortality risk.

## 1. Introduction

Reoperations after cardiac surgery pose a significant challenge for surgeons, as they are associated with a longer cardiopulmonary bypass time (CPB), the complexity of underlying pathology and comorbidities with high risk for unintentional injury during thoracic re-entry, and, consequently, poorer survival [1,2]. In the past, aortic operations were considered high-risk procedures and were often performed only in emergency situations. After years of practice, the operation on the aorta became safer and standards were established [3,4]. The awareness of aortic causes of emergent situations and the improved detection of aortic diseases led to an increase in the number of aortic operations. A higher number of patients survive the initial procedure and may require a second intervention [5]. The main reasons for reoperation stem from the progression of the aortic disease, structural failures in the graft, like degeneration, or the appearance of pseudoaneurysms. Some patients need reoperation due to complications from the initial surgery, recurrent aortic dissection, or endocarditis [6,7]. Due to standardized follow-up examinations with advanced techniques, which are performed regularly, the decision to undergo reoperation can be made early. Also, dissections and pseudoaneurysms are detected routinely, and interventions can be performed in a controlled setting rather than a salvage setting. The patients awaiting reoperation show increased risk factors like multiple comorbidities, advanced age, and other cardiac diseases. Advanced imaging techniques like 3D reconstructions enable us to preoperatively plan for a given surgical procedure. Operative techniques have been refined over recent decades and now provide a wider range of abilities for different anatomic surgical challenges [8]. Despite the progress made, reoperation on the aorta remains a challenging intervention that requires experience and a high surgical level of expertise [9]. In this study, we aimed to assess long-term outcomes and identify risk factors for mortality in patients undergoing aortic arch reoperations

## 2. Materials and Methods

### 2.1. Patients and Data Collection

This retrospective observational, single-center study included all consecutive patients undergoing open surgical reoperation on the aortic arch between 1999 and 2023, covering a 24-year period. In total, 108 consecutive patients were included. Demographic information, clinical variables, operative details, and postoperative outcomes were systematically extracted from hospital records. The index operation was a procedure on the ascending or descending aorta and/or aortic root and/or an operation on the aortic valve. Patients with a history of sternotomy for other procedures not including the aorta, e.g., coronary artery bypass graft surgery and mitral valve surgery, were excluded from the analysis. Patients who underwent a subsequent reoperation involving the aortic arch were included in the present analysis. The primary indications for reoperation on the aortic arch were dissection, endocarditis, false aneurysm/rupture, or dilatation. Aortic dilatation requiring surgical interventions was defined based on the clinical guidelines applicable at the time of reoperation (1999–2023). The decision to operate was made by the treating surgeon in accordance with those standards. In general, the indication for surgical treatment was based on the location of the dilatation, the patient’s medical history, and relevant clinical risk factors [10]. At our institution at that time, surgical treatment was typically performed when the aortic arch diameter reached ≥55 mm, or ≥50 mm in patients with a bicuspid aortic valve. Lower thresholds were considered in specific cases, such as with a small body surface area, with rapid aneurysm progression, according to patient preference, or when a concurrent proximal aortic procedure was planned. Precise measurements of ascending or descending aortic diameter were not consistently documented in medical records. Being under 18 at reoperation and transcatheter aortic valve replacement during the initial procedure were the exclusion criteria for the study. The principal outcome was the incidence of mortality over the observation time of the study. Additional outcomes of interest included cardiac re-reoperation, new aortic type B dissection, infective endocarditis, readmission due to cardiac cause, the need for temporary mechanical circulatory support (tMCS), coronary intervention, and neurological complications (intrahospital neurovascular complications including intracranial bleeding, stroke or transient ischemic attack and delirium, or pacemaker implantation. The data were acquired through patient reports, data on the intensive care unit (ICU) and intrahospital stay, operation reports, written follow-up reports with the patient’s primary care physicians, and telephone follow-up with patients and relatives.

### 2.2. Statistical Analysis

Categorical data were presented as absolute numbers and percentages. Continuous variables were reported as medians with interquartile ranges (IQRs) or means with standard deviations. Kaplan–Meier survival analysis was performed to estimate overall survival following reoperation. Overall survival rates were compared between subgroups stratified for the number of previous sternotomies, the urgency of the procedure (elective vs. non-elective), and gender using the log-rank test. Median survival times and survival rates at specific time points were reported where appropriate. Survival analyses were performed using Cox proportional hazard regression models. Initially, univariate Cox regression analyses were conducted to identify potential predictors of mortality. Variables with a *p*-value < 0.10 in the univariate analysis were considered for inclusion in the multivariate models. To minimize the risk of overfitting, given the limited number of events (51 patients with the primary outcome of mortality), we excluded variables with strong clinical correlations to other significant predictors. Variables were initially tested in nested multivariate Cox regression models. Gender and age were included in all models. In the final multivariate model, all significant predictors in the univariate analysis were tested. Results were expressed as hazard ratios (HRs) with corresponding 95% confidence intervals (CIs). Receiver operating characteristic (ROC) curve analysis was conducted to evaluate the prognostic significance of age. A cut-off value of 57.5 years, derived from the ROC analysis, was used to perform a sensitivity analysis comparing younger and older patients to further assess the impact of age on mortality. *p* values below 0.05 were considered significant. Statistical analysis was performed using SPSS Version 26 (IBM Corp., Armonk, NY, USA) and R Version 4.4.3 (R Core Team 2024, Vienna, Austria).

## 3. Results

### 3.1. Patient Characteristics

A total of 108 patients were included in this study. The mean patient age was 56 ± 14 years, and 75% (81/108) were male. Patient characteristics are shown in Table 1.

The mean age was 56 ± 14 years; 75% (81/108) were male and presented with NYHA (New York Heart Association) class III–IV at reoperation (65.3%, 47/72). The mean EuroSCORE II was 5.12 (2.77–10.22).

### 3.2. Index Operation

This analysis included patients with a history of sternotomy with an index operation at the aorta and/or an operation on the aortic valve. We found that 8 out of 106 patients (7.5%) had undergone an aortic arch or descending aortic procedure during their initial operation. Isolated aortic valve replacement was performed in 27.4% (29/106) of patients, isolated supracoronary ascending aortic replacement in 15.1% (16/106), combined aortic valve and aorta ascendens replacement in 10.4% (11/106), and root replacement in 24.5% (26/106). This heterogeneous distribution of index procedures reflects both the complexity of the patient population and the long inclusion period, spanning more than two decades. The most common diagnosis at the time of the index operation was type A aortic dissection or rupture (45.7%, 48/105), followed by valvular disease (31.4%, 33/105) (Table 2). A bicuspid aortic valve was present in 16.9% (13/77) of patients, and connective tissue disease was diagnosed in 12.0% (13/108). Since the study spanned two decades and many patients underwent their index surgery at other institutions, data on arch dimensions at the time of the initial operation and the rationale for not performing arch replacement were unavailable.

### 3.3. Reoperation

The mean time between the initial operation and reoperation was 6.84 years (1.61–14.94 years). The primary indication for reoperation was aortic arch dilation (47.2%, 51/108). During reoperation, all patients underwent a partial or total arch replacement, with the majority (61.1%, 66/108) receiving a partial arch replacement and the remainder undergoing total arch replacement using the island technique. In addition, 52.3% (56/107) of patients received a concomitant ascending aortic replacement, 33.0% (35/106) underwent a procedure on the descending aorta, and 29.6% (32/108) required a combined arch and root replacement. The second most common indication for arch reoperation was dissection (40.7%, 44/108). Among the 44 patients who underwent reoperation due to dissection, 40 had acute type A dissections and 6 had type B dissections, including 4 cases of isolated type B dissection. Nearly half of the patients underwent surgery in an urgent or salvage setting. The reoperation data are shown in detail in Table 3.

### 3.4. Perioperative and In-Hospital Outcomes

Based on the operative data presented in Table 4, the mean operation duration was 389 (320–489) min, with a cardiopulmonary bypass (CPB) time of 210 (160–248) min, an aortic clamp time of 103 (71–143) min, and a cerebral perfusion time of 38 (18–70) min. Patients remained in the ICU for an average of 6 (2–11) days, while the median total hospital stay was 15 (10–24) days. In two patients, a second cardiopulmonary bypass run, and a second aortic cross-clamping were necessary. Both patients were alive at follow-up.

A detailed analysis of postoperative events during the intrahospital stay was conducted. A transfusion due to bleeding, as defined by the BARC criteria, was required in 15.7% (17/108) of patients, all of whom subsequently required reintervention due to tamponade or hemothorax. Additionally, 4.6% (5/108) of patients required temporary mechanical circulatory support, while 19.4% (21/108) experienced neurovascular complications. A summary of postoperative complications is provided in Table 5.

### 3.5. Outcomes

**Figure 1:**
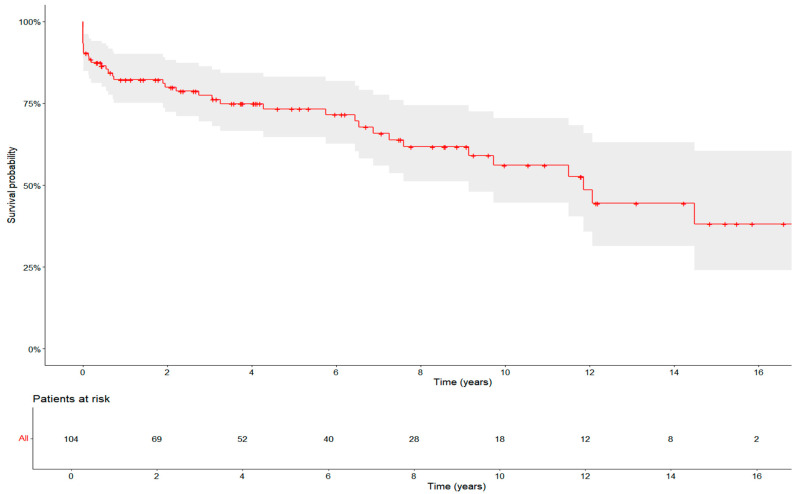
Survival over the course of observation time: Kaplan–Maier curve for overall mortality after reoperation on aortic arch.

The principal outcome was mortality. The median follow-up was 3.91 (0.82–8.58) years, with a maximum of 22.54 years. The 30-day mortality was 10.6% (11/108). In-hospital mortality was 7.4% (8/108), while 25% (27/108) of patients died after hospital discharge. The overall survival rates were 82% (±7%) at 1 year, 73% (±8%) at 5 years, and 56% (±14%) at 10 years (Figure 1). The leading causes of death included cardiovascular disease (11.1%, 12/108), bleeding (8.3%, 9/108), and non-cardiac conditions such as sepsis (7.4%, 8/108). In 12% (13/108) of cases, the cause of death remained unknown. Cardiovascular mortality was defined as death resulting from myocardial infarction, sudden cardiac death, heart failure, cardiogenic shock, or stroke, with cardiogenic shock due to pump failure being the most common cause (Supplementary Table S1). A cardiac re-reoperation following the initial reoperation was required in 17.6% (19/108) of patients, with a median time to re-reoperation of 1.80 (0.28–9.35) years. Indications for reoperations included type B dissection, false aneurysm, endocarditis, and hemodynamically relevant valvular or paravalvular insufficiency of the aortic valve.

Other outcomes comprised major postoperative complications, including bleeding, new type B aortic dissection or rupture, infective endocarditis, readmission due to cardiac causes, cardiovascular (coronary revascularization) and neurolovascular events (stroke), pacemaker implantation, and tMCS implantation. Readmission due to a cardiac cause was necessary for 34.3% (37/108) of patients. Among the discharged patients, 3.7% (4/103) developed infective endocarditis, all of whom required surgical intervention. Stroke after discharge was diagnosed in 2.8% (3/108) of patients. A detailed summary of the outcomes of the current study is provided in Supplementary Table S1.

### 3.6. Risk Factor Analysis

We performed a univariate analysis to identify risk factors associated with mortality. Several variables emerged as significant predictors, including (a) preoperative factors such as age (HR = 1.04, *p* ≤ 0.01) and a history of CAD (HR = 2.90, *p* = 0.01), (b) the need for a salvage operation (HR = 6.41, *p* ≤ 0.01), c) perioperative factors like the duration of the reoperation (HR = 1.00, *p* = 0.04), bypass time (HR = 1.01, *p* = 0.01), reperfusion time (HR = 1.01, *p* = 0.02), and ventilation time (HR = 1.03, *p* ≤ 0.01), and, finally, postoperative complications like bleeding requiring transfusion or intervention (HR = 3.98, *p* ≤ 0.01) and the need for tMCS (HR = 3.58, *p* = 0.04). A detailed summary of the univariate analysis results is provided in Supplementary Table S2.

We performed a nested Cox proportional hazard model using variables that were statistically significant in the univariate analysis. After multivariate adjustment, age (HR = 1.04, *p* ≤ 0.001), the need for a salvage operation (HR = 5.38, *p* = 0.01), a prolonged ventilation time (HR = 1.08, *p* = 0.04), and bleeding requiring transfusion or reintervention (HR = 3.76, *p* = 0.03) at reoperation were associated with a worse prognosis. The Cox proportional hazard model is presented in Table 6 and Table 7.

We conducted an additional subgroup analysis to examine differences in overall survival among specific groups of interest. These groups included (a) the electiveness of reoperation (elective vs. non-elective) and (b) the history of sternotomies (patients with 1 prior sternotomy vs. ≥2 sternotomies). Supplementary Figure S1 presents the Kaplan–Meier curves for each subgroup. Although patients undergoing elective reoperation had a longer mean survival of 13.0 years (95% CI: 9.7–16.2) compared to 9.1 years (95% CI: 7.0–11.2) for those undergoing non-elective reoperations (log-rank *p* = 0.19), the difference was not statistically significant. Similarly, patients with a history of two or more sternotomies had a worse mean survival of 7.1 years (95% CI: 3.8–10.4) compared to 13.1 years (95% CI: 10.5–15.7) for those with only one prior sternotomy (Log-rank *p* = 0.07), though this difference also did not reach statistical significance (Supplementary Figure S1). A survival analysis stratified for subgroups of clinical interest (gender, number of sternotomies, and electiveness of procedure at reoperation) revealed no significant differences on survival.

Age was a significant predictor of outcomes following reoperation on the aortic arch. Based on the optimal Youden index for mortality prediction, a cut-off value of 57.5 years effectively stratified the risk of death in the ROC analysis (area under the curve 0.692, *p* < 0.01), with a sensitivity of 61% and a specificity of 59.7% (Supplementary Figure S3).

Based on the findings of the ROC analysis with a cut-off value of 57.5 years, we performed a sensitivity analysis, stratifying the patients into those below and those above this threshold. In patients older than 57.5 years, the sensitivity analysis identified a history of myocardial infarction (HR = 5.81, *p* = 0.03), endocarditis as the indication for reoperation (HR = 15.49, *p* = 0.02), the need for a salvage operation (HR = 8.48, *p* = 0.02), the reoperation duration (HR = 1.00, *p* = 0.04), the bypass time (HR = 1.00, *p* = 0.03), undergoing a descending aortic procedure at reoperation (HR = 3.68, *p* = 0.01), and postoperative complications such as gastrointestinal complications (HR = 11.99, *p* = 0.03), bleeding (HR = 5.00, *p* ≤ 0.01), and the need for tMCS (HR = 80.23, *p* ≤ 0.01) as strong predictors of mortality.

## 4. Discussion

The available data on aortic arch reoperations are sparse and often imprecise. Many studies include interventions on other parts of the aorta or heart, making direct comparisons challenging [4,11,12]. The anatomical complexity of the aortic arch and the variability in surgical approaches further contribute to the difficulty in drawing definitive conclusions. Additionally, differences in patient selection criteria, surgical techniques, and institutional expertise introduce further variability to reported outcomes. In this institutional study, we distinctly differentiate aortic arch reoperations from other aortic interventions, ensuring clearer and more comparable results. In other studies, the long-term survival rates following reoperation were 81%, 67%, and 56% at one, five, and ten years, respectively [11,13]. In our cohort, survival at these time points was 82%, 73%, and 56%. These findings suggest that reoperation can be performed safely, as demonstrated across multiple studies. A key finding of our study was that mortality was primarily driven by operative and in-hospital mortality, with a 30-day mortality rate of 10.6%. However, prognosis after discharge was favorable, and the risk of cardiac re-reoperation was acceptable (17.6%). These results indicate that despite the complexity of aortic arch reoperations, the procedure can achieve satisfactory long-term outcomes when performed in experienced centers [14].

### 4.1. Risk Factors for Mortality

Regarding risk factors for adverse outcomes, we validated several well-known predictors within our patient cohort. Patients undergoing repeat cardiac surgery often present with a higher risk profile, particularly those with a history of coronary artery disease (CAD), older age, and multiple comorbidities. As survival rates following initial aortic surgery improve, the patient population requiring reoperation is progressively aging. Elderly patients generally present a higher surgical risk due to reduced physiological resilience and an increased burden of comorbidities [15]. However, a critical question remains: At what age does aortic arch reoperation become too high-risk?

In our ROC analysis, we identified a cut-off age of 57.5 years as being associated with worse outcomes. Similarly, the study by Risteski et al. identified age as the only independent predictor of mortality [16]. Sensitivity analysis confirmed several strong predictors of mortality, including a history of myocardial infarction or endocarditis as the indication for reoperation (HR = 15.49, *p* = 0.02), the need for a salvage procedure (HR = 8.48, *p* = 0.02), prolonged reoperation duration (HR = 1.00, *p* = 0.04), an increased cardiopulmonary bypass time (HR = 1.00, *p* = 0.03), and undergoing a descending aortic procedure during reoperation (HR = 3.68, *p* = 0.01). Postoperative complications were also significant predictors, particularly gastrointestinal complications (HR = 11.99, *p* = 0.03), severe bleeding (HR = 5.00, *p* ≤ 0.01), and the need for tMCS (HR = 80.23, *p* ≤ 0.01). Although age is an established risk factor that cannot be modified preoperatively, our findings support the decision-making process regarding the feasibility of elective reoperation. In patients over 60 years, reoperation should primarily be considered in cases of imminent life-threatening risk, with alternative approaches explored when possible. One such alternative is endovascular repair for aneurysmal dilation. However, endovascular techniques for aortic arch repair remain in their early stages and carry a high risk of neurological complications [12]. Despite these risks, endovascular approaches may become a viable option for high-risk patients in the future, potentially avoiding the need for repeated sternotomy.

The decision to extend aortic replacement during the initial surgery in acute type A aortic dissection remains critical. Zierer et al. identified the non-resection of the primary tear as an independent predictor of late reoperation, leading them to adopt an aggressive hemiarch replacement strategy when the tear was within the arch, while reserving total arch replacement for chronic dissections with aneurysmal dilation [17,18]. Also, the importance of initial surgical extent is further underscored by the findings of Rylski et al., who studied outcomes after type A dissection in patients with Marfan syndrome. They found that secondary arch surgery was required only in patients who initially underwent hemiarch replacement, suggesting that more extensive initial arch repair may reduce the need for late reinterventions [19]. Other investigators focused on a specific surgical indication—dissection—in patients (n = 50) with prior proximal aortic surgery and developed an alternative approach involving reoperative total arch repair using a trifurcated graft, elephant trunk, and selective antegrade cerebral perfusion. Their technique emphasized a staged repair strategy, yielding favorable outcomes [20]. This novel approach, however, was not applied in our study population, and direct comparisons cannot be made. Our findings further highlight the perioperative risks associated with reoperation, particularly the challenges posed by repeated sternotomies. To determine whether a more extensive initial arch replacement should be routinely performed, detailed data on aortic arch size at the time of the index procedure are essential. However, due to the long study period and the fact that many patients underwent their initial surgery at other institutions, such data are often unavailable. These limitations underscore the need for a more proactive approach during the initial operation, as more extensive repair may reduce the likelihood of late reoperation and its associated risks. Advanced preoperative imaging is essential in the better intraoperative management of scar tissue adhesions and in assessing the integrity of the aorta [21]. Consequently, we routinely perform preoperative computed tomography (CT) in all patients. Additionally, patients who underwent their initial aortic surgery at our institution due to dissection or dilation are monitored postoperatively with follow-up CT scans to detect re-dissections or dilations at an early stage. This approach aligns with Gaudino’s meta-analysis on aortic reoperations, which emphasizes the importance of long-term surveillance following proximal aortic surgery [3]. Implementing an imaging surveillance protocol allows for the earlier detection of indications for reoperation and may improve overall outcomes [22].

### 4.2. Postoperative Bleeding and Mortality

Postoperative bleeding remains a significant risk factor for mortality following aortic arch reoperation. In our study, bleeding requiring transfusion—classified by the Bleeding Academic Research Consortium (BARC) criteria—was strongly associated with increased perioperative morbidity and mortality. Two primary mechanisms contribute to postoperative bleeding: technical surgical challenges and coagulopathy, often exacerbated by preoperative anticoagulation therapy. In elective cases, the proper management of anticoagulation is crucial in minimizing perioperative bleeding risk. When feasible, anticoagulation should be temporarily discontinued or bridged with alternative agents to optimize coagulation status before surgery. In emergency cases, the close intraoperative monitoring of bleeding parameters and the timely correction of coagulopathy can help mitigate excessive blood loss [23]. Additionally, in cases where bleeding necessitates surgical re-exploration, the need for a second intervention significantly increases perioperative mortality. Thus, meticulous hemostasis during the initial procedure is essential in reducing postoperative complications and improving long-term survival.

### 4.3. Limitations

This study was conducted retrospectively at a single institution. Moreover, it was carried out at a high-volume, highly specialized center with extensive expertise in aortic surgery and postoperative intensive care. The study population was heterogeneous with respect to the index procedures, a characteristic commonly seen in all studies addressing aortic arch reoperations. In our study, this heterogeneity may also be explained by the long observation period and the complex clinical profiles of the patients. To validate our findings and enhance their generalizability, larger multicenter studies with broader patient populations are required. Additionally, surgical techniques have advanced over time, and perioperative management standards have evolved, which may influence outcomes and should be considered in future research.

## 5. Conclusions

Reoperations on the aortic arch remain a complex but necessary operation in patients with progressive aortic arch disease. Despite the technical challenges, our institutional data demonstrate long-term survival outcomes comparable with previously published studies, underscoring the safety and efficacy of these procedures when performed in experienced centers. Identifying high-risk patients, optimizing preoperative assessment, and implementing meticulous surgical techniques are key in improving outcomes. While alternative approaches such as endovascular repair may become more viable in the future, open surgical reoperations remain the gold standard for treating aortic arch pathology in appropriately selected patients. Continued advancements in imaging, surgical techniques, and perioperative care will further enhance the safety and success of these challenging procedures.

## Figures and Tables

**Table 1:** Baseline characteristics.

Variable	
Male n/N (%)	81/108 (75%)
Age (years)	56 ± 14
BMI (kg/m^2^)	26.50 ± 4.50
Arterial hypertension	38/91 (41.8%)
Diabetes mellitus	8/105 (7.7%)
Dyslipidemia	30/105 (28.6%)
History of CAD	15/99 (15.2%)
Smoking history	37/97 (38.1%)
History of myocardial infarction	7/104 (6.7%)
History of neurovascular events	17/104 (16.3%)
Chronic lung disease	12/69 (17.4%)
Peripheral artery disease	5/104 (4.8%)
Chronic kidney disease	14/103 (13%)
Creatinine (mg/dL)	1.02 ± 0.35
Chronic liver disease	3/108 (2.8%)
EuroSCORE II (%)	5.12 (2.77–10.22)
LVEF (%)	60 (55–62)
LVEF	
Normal	59/69 (85.5%)
Mild	6/69 (8.7%)
Moderate	2/69 (2.9%)
Severe	2/69 (2.9%)
NYHA	
I	6/72 (8.3%)
II	19/72 (26.4%)
III	29/72 (40.3%)
IV	18/72 (25.0%)
Bicuspid aortic valve	13/77 (16.9)
Connective tissue disease	13/108 (12.0%)
Resection of coarctation of the aorta or commissurotomy	7/108 (6.5%)
Number of Sternotomies before Re-OP	
1	89/108 (82.4%)
2	17/108 (15.7%)
3	2/108 (1.9%)

BMI, body mass index; CAD, coronary artery disease; LVEF, left ventricular ejection fraction; NYHA, New York Heart Association.

**Table 2:** Index operation data.

Procedure at previous operation	
Aortic valve procedure	29/106 (27.4%)
Supracoronary ascending aortic replacement	16/106 (15.1%)
Aortic valve and aorta ascendens replacement	11/106 (10.4%)
Root replacement	26/106 (24.5%)
Arch replacement or stenting of descending aorta	8/106 (7.5%)
Resection of coarctation of aorta	3/106 (2.8%)
Combination	13/106 (12.3%)
Concomitant procedures with previous operation	9/106 (8.5%)
Diagnosis at previous operation	
Dilatation	9/105 (8.6%)
Rupture/Dissection	48/105 (45.7%)
Endocarditis	2/105 (1.9%)
Valvular disease	33/105 (31.4%)
Valvular disease and dilatation	13/105 (12.4%)
Prosthetic valve type in aortic position at previous operation	
Biological	24/104 (23.1%)
Mechanical	34/104 (32.7%)

**Table 3:** Perioperative data.

Diagnosis at reoperation	
Dilatation	51/108 (47.2%)
Rupture/false aneurysm	12/108 (11.1%)
Dissection	44/108 (40.7%)
Endocarditis	12/107 (11.2%)
Intervention on aortic arch at reoperation	
Total arch replacement	36/108 (33.3%)
Partial arch replacement	66/108 (61.1%)
Other *	6/108 (5.6%)
Aorta descendens procedure	35/106 (33.0%)
Supracoronary ascending aortic replacement	56/107 (52.3%)
Combined procedure on aortic root and arch at reoperation	32/108 (29.6%)
Prosthetic valve type in aortic position at reoperation	
Biological	23/108 (21.3%)
Mechanical	19/108 (17.6%)
Concomitant procedures at reoperation	82/108 (75.9%)
Concomitant procedures at reoperation	
Aorta	60/108 (55.6%)
CABG	6/108 (5.6%)
Mitral valve	2/108 (1.9%)
Tricuspid valve	1/108 (0.9%)
Combination	10/108 (9.3%)
“UFO” procedure	1/108 (0.9%)
Subclavian Bypass	2/108 (1.9%)
Urgency of reoperation	
Elective	56/108 (51.9%)
Urgent	28/108 (25.9%)
Emergency	17/108 (15.7%)
Salvage	7/108 (6.5%)

* The ‘Other’ category (5.6%) includes hybrid arch procedures such as arch reconstruction using patches, isolated left subclavian artery reconstructions, and complex interventions involving stent extension or endoleak closure not classifiable as standard total or partial arch replacements.

**Table 4:** Operative data on reoperation.

Operative Data	
Duration (min)	389 (320–489)
Bypass Time (min)	210 (160–248)
Aorta clamp time (min)	103 (71–143)
Reperfusion time (min)	65 (45–87)
Second period on heart–lung machine	2/108 (1.9%)
Second clamping of aorta	2/108 (1.9%)
Ventilation time (days)	1 (1–4)
ICU stay (days)	6 (2–11)
Hospital stay (days)	15 (10–24)

ICU, intensive care unit.

**Table 5:** Postoerative Complications.

Postoperative Complications	
Gastrointestinal bleeding requiring transfusion	4/108 (3.7%)
Neurovascular complications *	21/108 (19.4%)
Delirium	8/108 (7.4%)
Intervention PCI	1/108 (0.9%)
Stenting of descending aorta	5/108 (4.6%)
Bleeding requiring transfusion or intervention	17/108 (15.7%)
Tamponade or haematothorax	17/108 (15.7%)
Requiring tMCS	5/108 (4.6%)
Requiring pacemaker	7/108 (6.5%)

* Refers to stroke, transient ischemic attack, or intracranial bleeding. PCI, percutaneous coronary intervention; tMCS, temporary mechanical circulatory support.

**Table 6:** Survival analysis with proportional hazard models for the incidence of death: nested models.

Variable	Hazard Ratio	95% Confidence Intervals	*p*
Model 1: Medical history			
Male gender *	0.70	0.31–1.59	0.39
Age (years)	1.04	1.01–1.08	**0.01**
History of ≥2 sternotomies **	1.30	0.48–3.48	0.60
Creatinine (mg/dL)	1.72	0.61–4.86	0.31
EuroSCORE II(%)	1.01	0.94–1.07	0.86
Coronary artery disease *	1.51	0.59–3.85	0.39
Model 2: Periprocedural data			
Male gender *	0.66	0.29–1.46	0.30
Age (years)	1.05	1.02–1.08	**≤0.001**
Supracoronary ascending aortic replacement at reoperation	0.49	0.25–0.99	0.05
Procedure at descending aorta at reoperation	1.64	0.75–3.58	0.22
Reoperation diagnosis: dilatation	0.52	0.24–1.14	0.10
Reoperation diagnosis: false aneurysm	1.04	0.41–2.66	0.93
Salvage operation ***	5.38	1.59–18.22	**0.01**
Model 3: Operative times and postoperative complications			
Male gender *	0.69	0.32–1.49	0.35
Age (years)	1.04	1.01–1.07	**≤0.01**
Duration time (minutes)	1.00	0.99–1.00	0.36
Bypass time (minutes)	1.01	1.00–1.02	0.06
Reperfusion time (minutes)	1.00	1.00–1.01	0.35
ICU stay (days)	0.93	0.86–1.01	0.07
Ventilation (days)	1.08	1.00–1.17	**0.04**
Bleeding after reoperation *	3.76	1.10–12.86	**0.03**
Tamponade/hemothorax after reoperation *	0.99	0.26–3.77	0.99
tMCS at reoperation *	2.35	0.41–13.68	0.34

* Reference category, absence of characteristic; ** reference category: history of one sternotomy; *** reference category: elective, urgent, and emergency operation combined. ICU, intensive care unit; tMCS, temporary mechanical circulatory support. Bold formatting indicates statistical significance at *p* ≤ 0.05.

**Table 7:** Survival analysis with proportional hazard models for the incidence of death.

Variable	Hazard Ratio	95% Confidence Intervals	*p*
Age (years)	1.06	1.02–1.09	≤0.001
Salvage operation **	5.15	1.17–22.67	0.03
Bleeding after reoperation *	3.53	1.42–8.81	0.01

* Reference category, absence of characteristic; ** reference category: elective, urgent, and emergency operation combined,. Additionally adjusted for and found not significant: male gender, history of ≥2 sternotomies, EuroScore II, coronary artery disease, supracoronary ascending aortic replacement at reoperation, procedure in descending aorta at reoperation, reoperation diagnosis of dilatation, reoperation diagnosis of false aneurysm, salvage operation at reoperation, duration (minutes), bypass time (minutes), ICU stay (in days), ventilation (in days), tMCS at reoperation. ICU, intensive care unit; tMCS, temporary mechanical circulatory support.

## Data Availability

The data that support the findings of this study are available from the corresponding author upon reasonable request.

## Supplementary Materials

The following supporting information can be downloaded at: https://www.mdpi.com/article/10.3390/jcm14124087/s1. Table S1: Outcomes; Table S2: Univariate Analysis; Figure S1: Survival analysis on selected subgroups; Figure S2: Subgroup survival analysis for age; Figure S3: ROC-Analysis for age.
