# Supplementary material for: Over Two Decades of Experience in Aortic Arch Reoperations: Long-Term Outcomes and Mortality Risk Factors
Source: J Clin Med. 2025 Jun 10;14(12):4087. doi: 10.3390/jcm14124087 (PMC12193799; doi:10.3390/jcm14124087)
Supplement: Supplementary file 1 [file jcm-14-04087-s001.zip › jcm-3639149-supplementary.pdf]

Supplementary Materials:

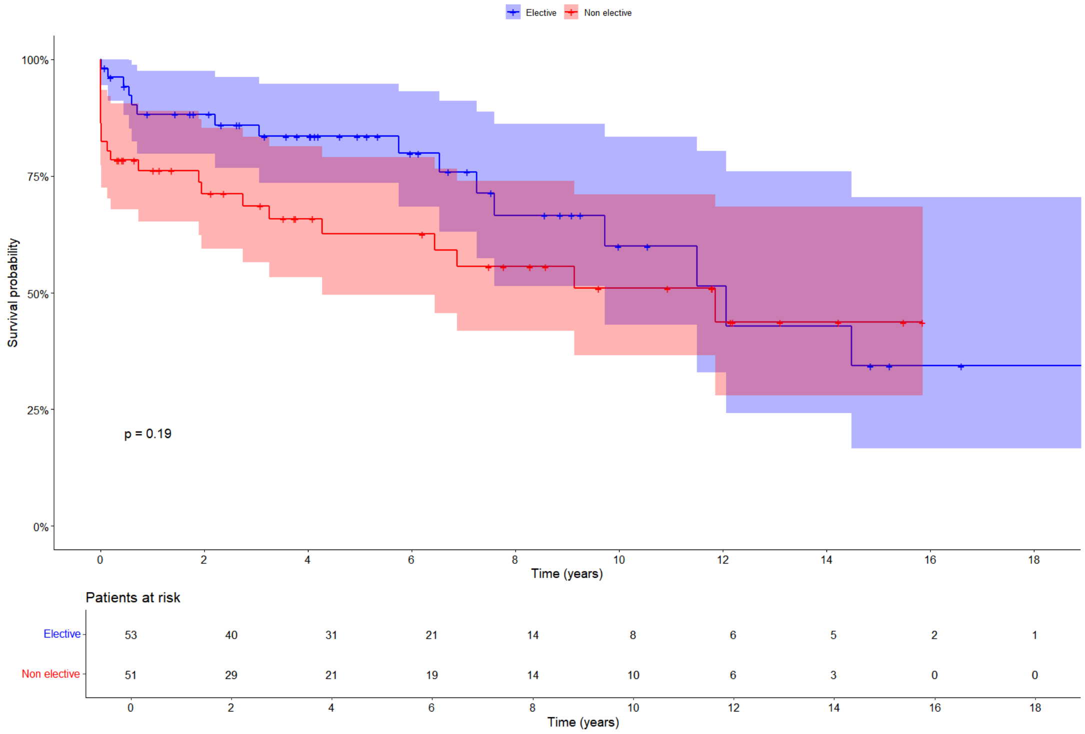

(A)

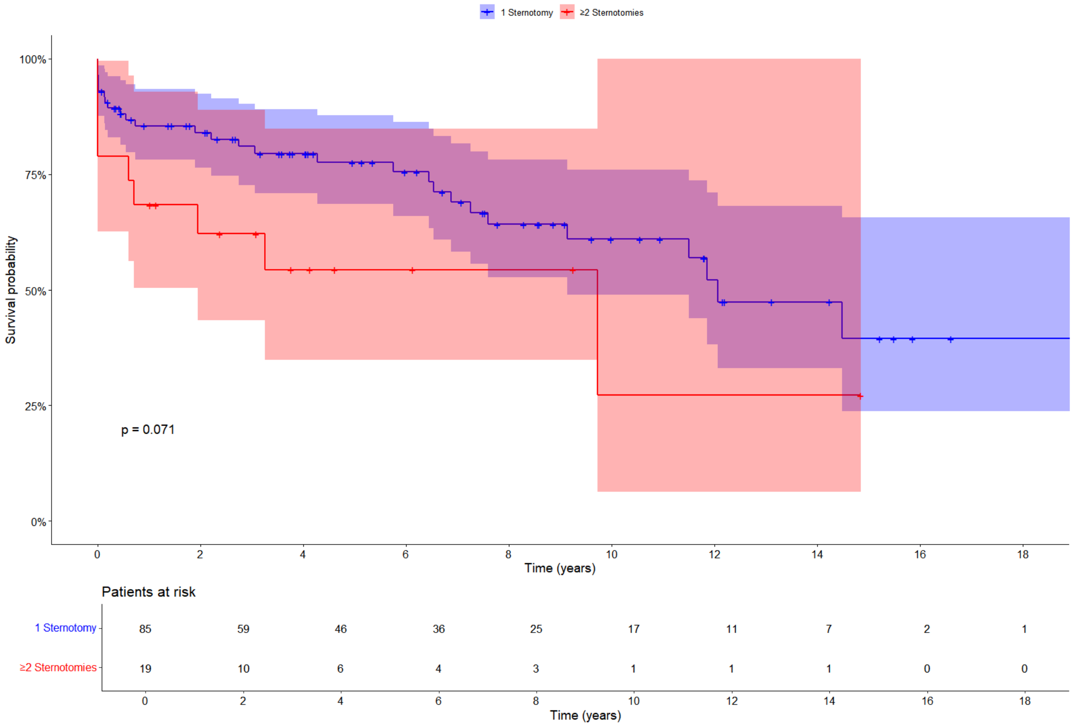

(B)

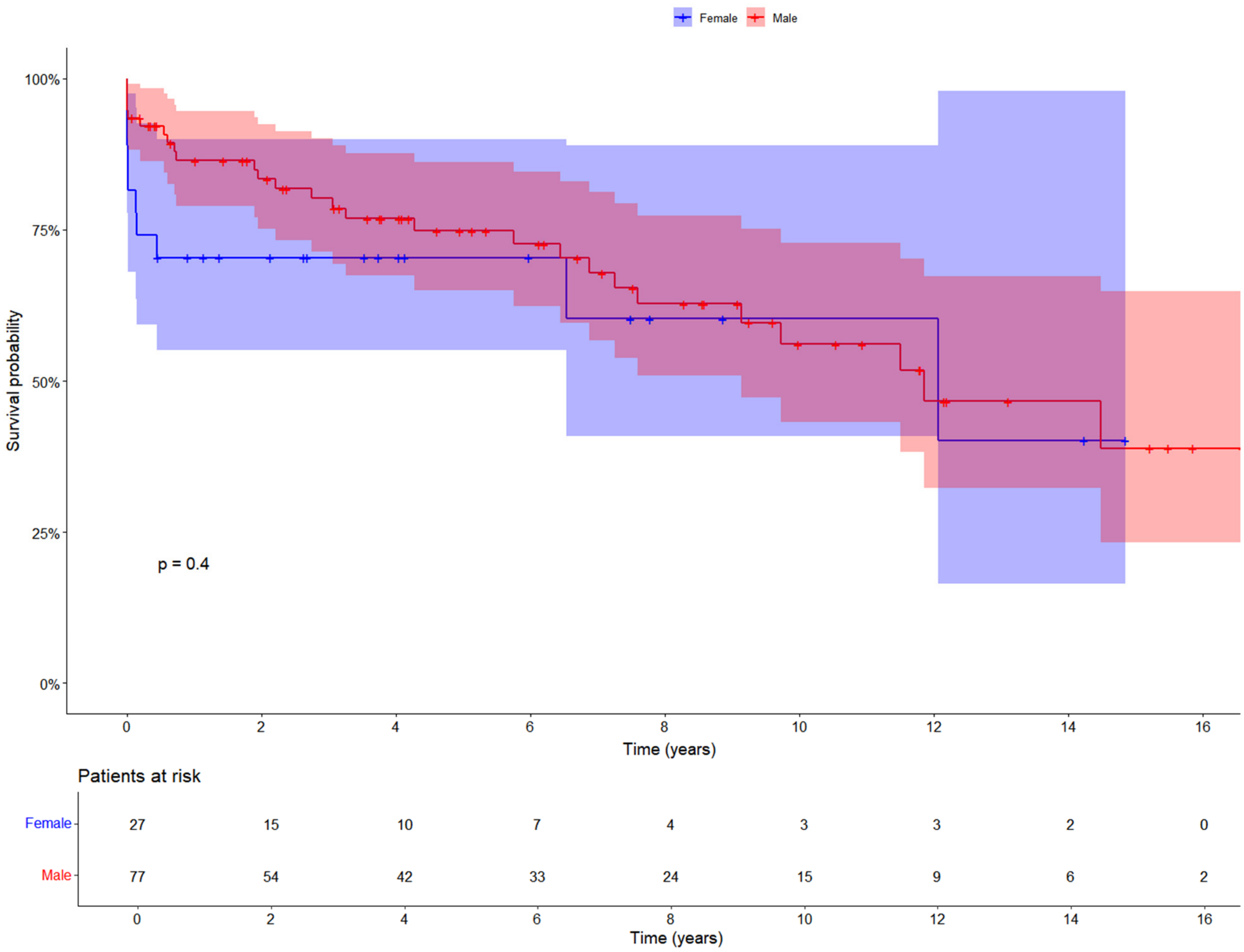

(C)

Figure S1. Survival analysis on selected subgroups. A. Survival analysis based on the electiveness of reoperation on the aortic arch: Kaplan-Meier curves demonstrating the survival over 16 years stratified for electiveness of re-operation (mean survival for elective re-operation 13 (95%CI: 9.7-16.2 ; blue color) years vs. 9.1 (95%CI: 7-11.2) years for non-elective reoperations; Log rank p =0.19 ; red color)**. B. Survival analysis stratified for the number of previous sternotomies**: Kaplan-Meier curves demonstrating the survival over 16 years comparing patients with history of 1 sternotomy (blue color) to patients with a history of 2 or more sternotomies (red color ; mean survival 13.1 (95%CI: 10.5-15.7) vs. 7.1 (95%CI: 3.8-10.4) years, respectively; Log rank p =0.07). **C. Survival analysis stratified for gender:** Kaplan-Meier curves demonstrating the overall survival over 16 years for female gender compared (blue color) to male gender (red color) (mean survival 9.1 (95%CI: 6.4-11.8) vs. 12.8 (95%CI: 10.1-15.5) years, respectively; Log rank p =0.40). Overall survival was defined as the time in years from reoperation on aortic arch until the end of follow up (September 2023) or the incidence of death.

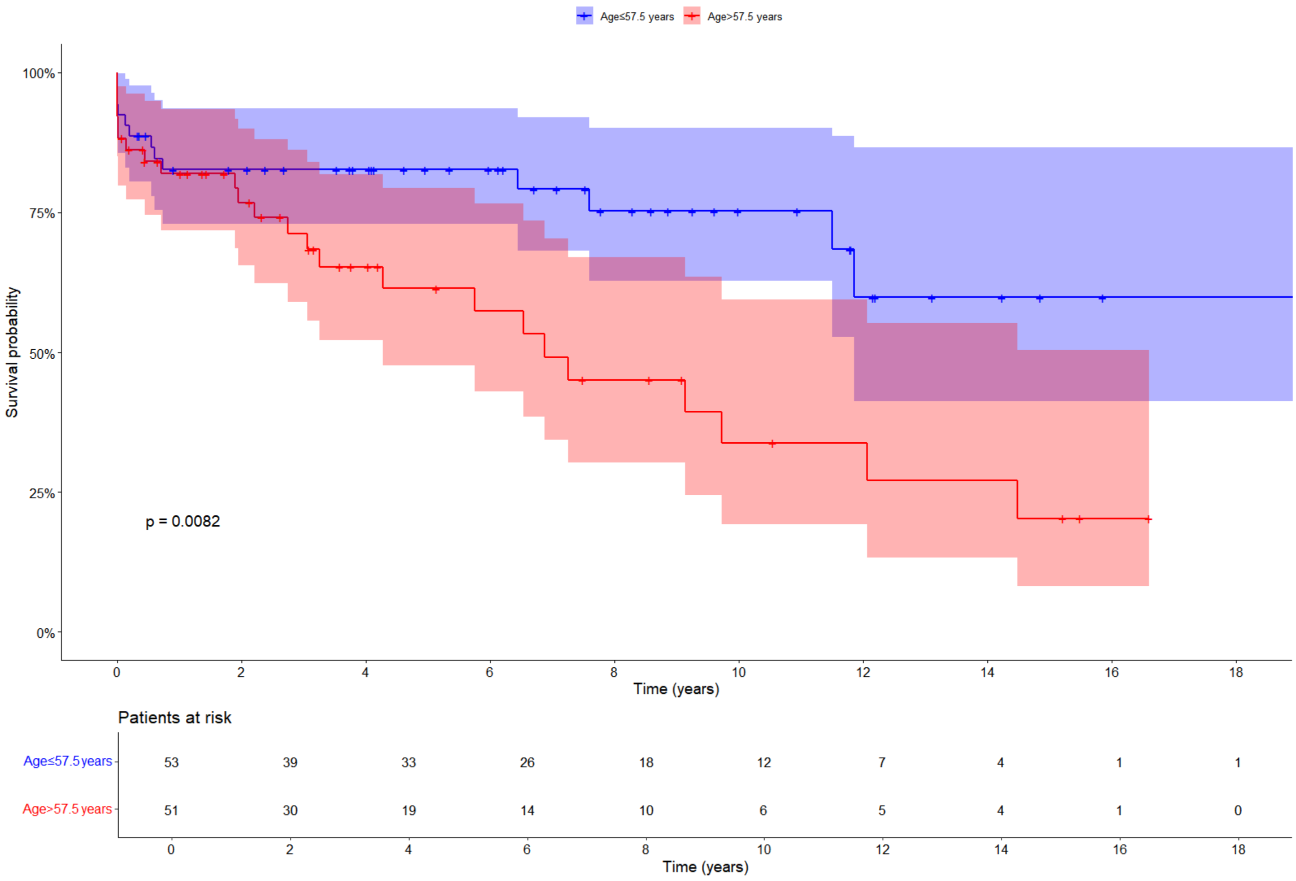

Figure S2. Subgroup survival analysis for age. Overall survival of patients who are ≤ 57.5 years old is significantly better as compared to those who are older than 57.5 years at the time of reoperation (mean survival 15.9 (95%CI: 12.7-19) vs. 7.9 (95%CI: 5.8-10) years, respectively; P ≤0.01; Kaplan-Meier survival analysis, log rank test). Overall survival was defined as the time in years from reoperation on aortic arch until the end of follow up (September 2023) or the incidence of death.

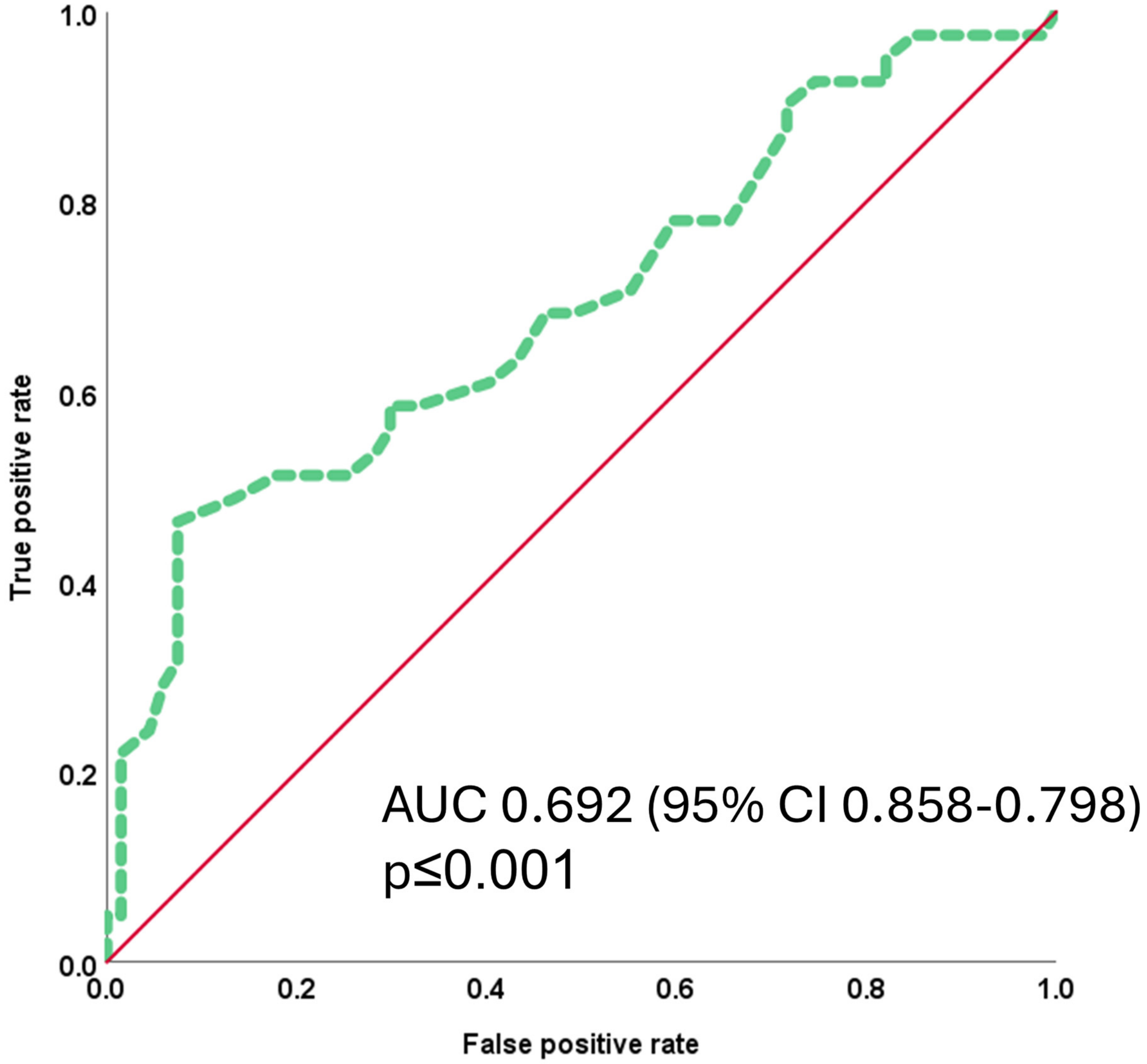

Figure S3. ROC-Analysis for age: Receiver operating characteristics analysis for age in years for the prediction of mortality during the observation time. The area under the curve (AUC) indicates significant potential prognostic value for age for predicting death in patients undergoing reoperation on aortic arch (AUC= 0.692; 95% CI: 0.585-0.798, p≤0.001). The cut-off value for age in years chosen with the best Youden index for the prediction of mortality was 57.5 years (sensitivity 61%, specificity 59.7%).

Table S1. Outcomes.

**Mortality**	**41/108 (38%)**
Cause of Death	
Cardiovascular	12/108 (11.1%)
Bleeding	9/108 (8.3%)
Non cardiac	8/108 (7.4%)
Unknown	13/108 (12%)
Time of Death	

In tabula	6/108 (5.0%)
Intrahospital	8/108 (7.4%)
Post-Discharge	27/108 (25.0%)
**Additional Outcomes after discharge**	
**Cardiac re-reoperation**	19/108 (17.6%)
Dissection type B/Rupture	15/93 (13.9%)
Pacemaker Implantation	2/108 (1.9)
Coronary intervention	2/108 (1.9%)
Stroke	3/108 (2.8%)
Bleeding requiring transfusion	9/108 (8.3)
Endocarditis	4/103 (3.7%)
Readmission due to cardiac cause	37/108 (34.3%)

Table S2. Univariate analysis.

Variable	HR	95 % CI			P value
Baseline characteristics					
Male n/N (%)	0.73	0.35	-	1.51	0.40
Age (years)	1.04	1.01	-	1.07	**≤0.01**
BMI (kg/m²)	1.03	0.94	-	1.14	0.50
Creatinine (mg/dl)	2.40	0.93	-	6.21	0.07
EuroSCORE II (%)	1.03	0.99	-	1.08	0.15
Arterial hypertension	0.76	0.35	-	1.65	0.49
Diabetes mellitus	1.54	0.54	-	4.37	0.42
Dyslipidemia	1.25	0.62	-	2.50	0.53
History of CAD	2.90	1.40	-	6.01	**0.01**
Smoking history	0.71	0.34	-	1.51	0.38
History of myocardial infarction	2.03	0.48	-	8.57	0.33
History of neurovascular events	0.78	0.30	-	2.02	0.61
Peripheral artery disease	0.52	0.07	-	3.85	0.53
Chronic lung disease	0.52	0.12	-	2.27	0.39
LVEF normal or not	0.97	0.28	-	3.31	0.96
NYHA ≥III	1.10	0.39	-	3.13	0.86
Bicuspid aortic valve	1.41	0.47	-	4.24	0.54
Connective tissue disease	0.47	0.14	-	1.54	0.21
Number of sternotomies before Re-OP ≥2	1.99	0.93	-	4.25	0.08
Data at previous operation					
Diagnosis at previous operation					
Dilatation	1.29	0.45	-	3.67	0.64
Rupture/Dissection	0.74	0.38	-	1.46	0.39
Endocarditis	3.11	0.42	-	23.16	0.27
Valvular disease	1.27	0.64	-	2.53	0.49
Valvular disease and dilatation	0.68	0.16	-	2.86	0.60
Procedure at previous operation					
Aortic valve replacement	1.01	0.49	-	2.10	0.97
Supracoronary ascending aortic replacement	1.32	0.58	-	3.02	0.51
Aortic valve and aorta ascendens replacement	0.30	0.04	-	2.18	0.23
Root replacement	0.87	0.41	-	1.83	0.72
Arch replacement or stenting of descending aorta	0.69	0.16	-	2.87	0.61
Resection of coarctation of the aorta	1.08	0.15	-	7.94	0.94
Combination	1.77	0.73	-	4.27	0.21
Concomitant procedures at previous operation	1.93	0.68	-	5.46	0.22

Prosthetic valve type in aortic position at previous operation					
Biological	1.13	0.51	-	2.50	0.76
Mechanical	0.72	0.35	-	1.47	0.37
Data at reoperation					
Diagnosis at reoperation					
Dilatation	0.49	0.25	-	0.99	0.05
Rupture/false aneurysm	2.08	0.91	-	4.75	0.08
Dissection	1.41	0.73	-	2.69	0.30
Endocarditis	1.49	0.58	-	3.85	0.41
Intervention on aortic arch at reoperation					
Total arch replacement	1.36	0.70	-	2.64	0.36
Partial arch replacement	0.65	0.33	-	1.25	0.19
Aorta descendens procedure	1.66	0.86	-	3.19	0.13
Valve type at reoperation					
Biological	0.70	0.27	-	1.81	0.46
Mechanical	1.10	0.48	-	2.50	0.83
Concomitant procedure at reoperation Yes or no	0.81	0.40	-	1.64	0.56
Concomitant procedures at reoperation					
Aorta	0.69	0.36	-	1.32	0.27
Other procedures	1.34	0.63	-	2.85	0.45
Combined procedure at aortic root and arch	1.22	0.60	-	2.48	0.58
Urgency of reoperation					
Elective	0.64	0.34	-	1.24	0.19
Urgent	0.78	0.35	-	1.71	0.53
Emergency	1.32	0.60	-	2.91	0.48
Salvage	6.41	2.44	-	16.83	**≤0.01**
Periprocedural Data					
Duration (min)	1.00	1.00	-	1.00	**0.04**
Bypass Time (min)	1.01	1.00	-	1.01	**≤0.01**
Aorta clamp time (min)	1.00	0.99	-	1.01	0.99
Single ischemia max (min)	1.00	0.99	-	1.01	0.98
Reperfusion time (min)	1.01	1.00	-	1.02	**0.02**
Ventilation time (days)	1.03	1.01	-	1.05	**≤0.01**
ICU stay (days)	1.02	1.00	-	1.05	0.07
Hospital stay (days)	1.00	0.98	-	1.03	0.80
Postoperative complications					
Gastrointestinal complication	2.12	0.65	-	6.94	0.22
Neurovascular complication	1.39	0.69	-	2.83	0.36
Delirium	0.79	0.11	-	5.83	0.81
Stenting of descending aorta (postoperative)	0.53	0.07	-	3.90	0.54
Bleeding requiring transfusion or intervention	3.98	1.95	-	8.15	**≤0.01**
Tamponade or haematothorax	2.53	1.14	-	5.60	**0.02**
Requiring temporary mechanical circulatory support	3.58	1.07	-	11.95	0.04
Requiring pacemaker	0.36	0.05	-	2.61	0.31

BMI: Body mass index, CAD: coronary artery disease, LVEF: left ventricular ejection fraction, NYHA: New York Heart Association
